# Data quality issues impede comparability of hospital treatment delay performance indicators

**DOI:** 10.1007/s12471-015-0708-3

**Published:** 2015-05-29

**Authors:** L.M. Verweij, J. Tra, J. Engel, R.A. Verheij, M.C. de Bruijne, C. Wagner

**Affiliations:** 1 The Netherlands Institute of Health Services Research (NIVEL), Utrecht, The Netherlands; 2 Department of Public and Occupational Health, EMGO + Institute, VU University Medical Center, Amsterdam, The Netherlands

**Keywords:** Acute coronary syndromes, Quality indicators, Data quality, Hospital information systems

## Abstract

**Aim:**

To assess the comparability of five performance indicator scores for treatment delay among patients diagnosed with ST-segment elevation myocardial infarction (STEMI) undergoing primary percutaneous coronary intervention in relation to the quality of the underlying data.

**Methods:**

Secondary analyses were performed on data from 1017 patients in seven Dutch hospitals. Data were collected using standardised forms for patients discharged in 2012. Comparability was assessed as the number of occasions the indicator threshold was reached for each hospital.

**Results:**

Hospitals recorded different time points based on different interpretations of the definitions. This led to substantial differences in indicator scores, ranging from 57 to 100 % of the indictor threshold being reached. Some hospitals recorded all the required data elements for calculating the performance indicators but none of the data elements could be retrieved in a fully automated way. Moreover, recording accessibility and completeness of time points varied widely within and between hospitals.

**Conclusion:**

Hospitals use different definitions for treatment delay and vary greatly in the extent to which the necessary data are available, accessible and complete, impeding comparability between hospitals. Indicator developers, users and hospitals providing data should be aware of these issues and aim to improve data quality in order to facilitate comparability of performance indicators.

## Introduction

Assessment of the quality of care by means of performance indicators is an integral part of modern day health care. Performance indicators are a tool in quality improvement and provide the government, physicians, patients, scientific society and insurance companies an indication of hospital performance, which is increasingly demanded [1]. As comparing performance indicator scores between hospitals can have major consequences, including lay press ranking lists and government and insurance company sanctions, performance indicator scores need to be comparable.

**Fig. 1:**
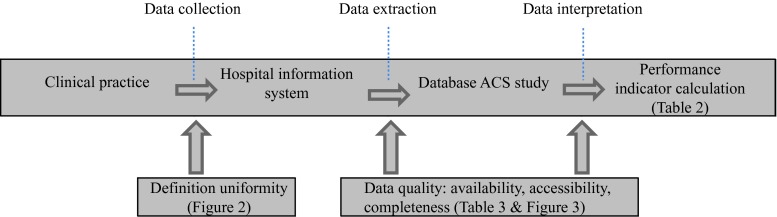
Comparability of data: flow from collection to interpretation.

There are several steps in the process that leads from an event happening in clinical practice to a performance indicator intended to measure the performance of a clinical practice regarding that event [2]. This process is illustrated in Fig. 1. Variations in any of these steps will lead to different performance indicator scores. Ideally, data recorded for performance indicators are based on sound clinical practice guidelines, in which the definitions and inclusion and exclusion criteria of the performance indicator are clear and unambiguous and then processed in a uniform way to calculate the performance indicator in a uniform way. In reality, however, definitions are far from unambiguous and data are recorded in a variety of ways, impeding comparability of indicators for external quality control [3, 4]. This means that users of performance indicators need to be aware of the possible impact of variations in definitions and quality of the data in terms of availability, accessibility and completeness [5, 6]. The more unambiguous the definitions and the higher the quality of the underlying data, the more likely the performance indicator scores will be accurate and consistent between hospitals [7].

For patients diagnosed with ST-segment elevation myocardial infarction (STEMI), international guidelines recommend timely invasive treatment by primary percutaneous coronary intervention (PCI), generally within 90 min of first medical contact [8, 9]. Delays in timely invasive treatment by PCI caused by, for example, residential distance rapidly decrease the benefits over alternative treatments [10, 11], while shortening delays has the potential to contribute to decreased heart failure and mortality [12, 13]. It is, however, unclear to what extent the treatment delay indicator scores are comparable between hospitals. This study therefore aims to investigate to what extent variations in definitions influence performance indicator scores. Moreover, we investigate to what extent the quality of data in terms of availability, accessibility and completeness influences performance indicator scores. We conclude by providing recommendations for improving comparability of performance indicator scores.

## Methods

### Patient data

Secondary data were used from two university hospitals and five tertiary teaching hospitals performing PCI participating in the acute coronary syndromes (ACS) program evaluation, within the larger national safety management program: ‘VMS safety management program’ [14].

Data from these seven hospitals were collected manually by six chart abstractors using standardised case report forms. All abstractors had a background in research and received instructions for the chart review procedures by JT and JE. The chart abstractors collected data by means of retrospective review of the medical records in electronic or paper-based medical, nursing or catheterisation laboratory records of patients discharged between 1 January and 31 December 2012. Each month, eligible records of patients discharged in the preceding month were selected from the hospital billing system using the diagnosis treatment combination code. To determine the STEMI population, chart abstractors first considered all the records of patients diagnosed with ACS for inclusion. Next, the chart abstractors checked whether the discharge letter confirmed the ACS diagnosis. When the discharge diagnosis was unclear, the record was discussed with a cardiologist or other attending physician working in the field of cardiology. Charts of patients with a treatment delay not exceeding 6 h were included in the study [15]. Charts of patients without a discharge diagnosis of STEMI, those not undergoing an acute PCI, patients with secondary ACS (e.g. due to anaemia), those undergoing elective procedures, patients with missing or uninformative charts and the charts of patients under the age of 18 years were excluded from the study. Chart abstractors signed a confidentiality agreement and all data were stored on a password protected network server of the VU University Medical Centre.

### Quality indicator definitions

**Fig. 2:**
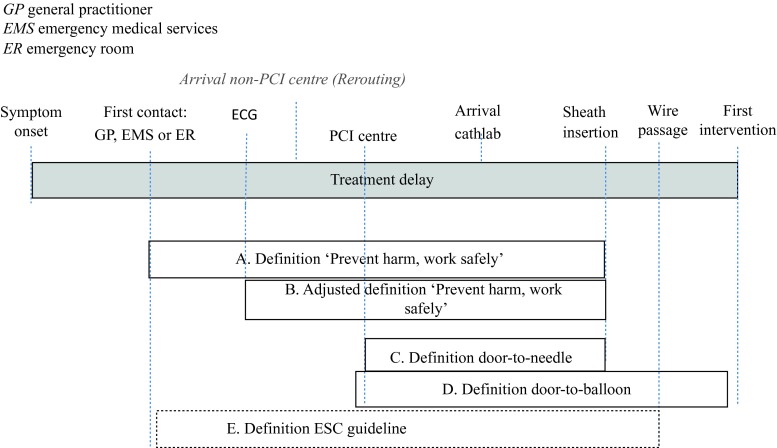
Delays from symptom onset to first intervention in patients with STEMI and five performance indicator definitions (A-E). *GP* general practitioner, *EMS* emergency medical services, *ER* emergency room.

Five definitions for the treatment delay indicator were derived from literature (Table 1 and Fig. 2): (A) The Dutch ‘VMS safety management program’ guidelines [14]; (B) The adjusted Dutch ‘VMS safety management program’ evaluation [14]; (C) The mean door-to-needle time [15]; (D) The door-to-balloon time (American ACC/AHA guidelines for the management of STEMI [9, 16]); and (E) The European Society of Cardiology guidelines for the management of STEMI [8]. In these five definitions, treatment delay was defined as: (A) PCI within 90 min of first medical/paramedical contact; (B) PCI within 90 min of first electrocardiogram (ECG); (C) the mean door-to-needle time (no threshold provided); (D) PCI within 90 min of hospital arrival, and (E) PCI within 90 min after first medical contact. The B definition is an adaption of the A definition, because the time of first medical/paramedical contact was not registered consistently in all PCI centres but the time of the first ECG was. Thus, for this study, treatment delay was defined as the time from first ECG to PCI. Noteworthy is further that indicator C asks for the *mean* door-to-needle time, illustrating that different organisations ask hospitals to register different information. Moreover, although none of the PCI centres registered the time of wire passage in the culprit artery, which is used by the ESC in the last definition, we provide this definition as an illustration because these guidelines provide the basis for the first and second definitions. For this study, we regarded the time from first ECG to PCI as the reference standard for pragmatic reasons. We emphasise that this definition is not a gold standard as there is no common gold standard for measuring treatment delay due to national and international differences and differences in perceptions of stakeholders. Moreover, the definitions are used for comparison reasons and not to conclude what the best definition is.

**Table 1:** Definitions for the performance indicator ‘treatment delay’.

**A.**	**VMSzorg.nl performance measure** (*source: VMS practice guide and VMS factsheet* [13])
*Definition*	Percentage of patients diagnosed with STEMI treated with PCI within 90 min of first medical/paramedical contact. we prefer to keep to the correct definition
*Numerator*	All patients undergoing PCI treatment within 90 min of first medical/paramedical contact. Medical/paramedical contact is defined as the general practitioner, emergency medical services or emergency department. PCI treatment is defined as the time of sheath insertion
*Denominator*	All patients diagnosed with STEMI
*Inclusion*	All patients diagnosed with STEMI
*Exclusion*	Patients with unstable angina or NSTEMI
**B.**	**Adjusted VMSzorg.nl performance measure** (*source:* [13])
*Definition*	Percentage of patients diagnosed with STEMI treated with primary PCI within 90 min of first ECG
*Numerator*	All patients diagnosed with STEMI treated with primary PCI within 90 min of first ECG. If patients developed a STEMI while being hospitalised for another illness or symptom, the time of the first ECG with ST-segment elevation in hospital was registered. Start of PCI is defined as the time of sheath insertion
*Denominator*	All patients diagnosed with STEMI treated with primary PCI
*Inclusion*	All patients with the diagnosis treatment combination code for ACS and discharge diagnosis of STEMI
*Exclusion*	STEMI patients undergoing pharmacological treatment or non-acute PCI (i.e. documented sub-acute or old infarction, ST-segment resolution on the electrocardiogram in combination with the absence of symptoms on admission); STEMI patients with > 6 h between ECG and PCI; patients with a secondary infarction (e.g. due to anaemia)
**C.**	**Dutch Health Care Inspectorate performance measure** (*source*: [14])
*Definition*	Mean door-to-needle time *Note: no threshold provided*
**D.**	**ACC/AHA performance measure** (*source*: [15])
*Definition 1*	Median time from hospital arrival to PCI in AMI patients with ST-segment elevation or LBBB on the ECG performed closest to hospital arrival time
*Definition 2*	AMI patients with ST-segment elevation or LBBB on the ECG closest to arrival time receiving primary PCI during the hospital stay with a time from hospital arrival to PCI of 90 min or less
*Numerator*	AMI patients whose time from hospital arrival to primary PCI is 90 min or less
*Denominator*	AMI patients with ST-segment elevation or LBBB on ECG who received primary PCI
*Inclusion*	Discharges with: An ICD-9-CM Principal Diagnosis Code for AMI AND PCI (ICD-9-CM Principal or Other Procedure Codes for PCI) AND ST-segment elevation or LBBB on the ECG performed closest to hospital arrival AND PCI performed within 24 h after hospital arrival
*Exclusion*	Patients less than 18 years of age Patients received in transfer from the inpatient, outpatient, or emergency department of another facility Patients given a fibrinolytic agent prior to PCI PCI described as non-primary by a physician/APN/PA Patients who did not receive PCI within 90 min and had a reason for delay documented by a physician, APN/PA (e.g. social, religious, initial concern or refusal, cardiopulmonary arrest, balloon pump insertion, respiratory failure requiring intubation)
**E.**	**ESC performance measure?** (*source*: [8])
*Description*	…a target for quality assessment is that primary PCI (wire passage) should be performed within 90 min after FMC in all cases. In patients presenting early, with a large amount of myocardium at risk, the delay should be shorter (60 min). In patients presenting directly to a PCI-capable hospital, the goal should also be to achieve primary PCI within 60 min of FMC. Although no specific studies have been performed, a maximum delay of only 90 min after FMC seems a reasonable goal in these patients. Note that these target delays for implementation of primary PCI are quality indicators and that they differ from the maximal PCI-related delay of 120 min, which is useful in selecting primary PCI over immediate thrombolysis as the preferred mode of reperfusion

*ACC/AHA* American College of Cardiology and American Heart Association, *ACS* acute coronary syndromes, *AMI* acute myocardial infarction, *APN*/*PA* advanced practice nurses/physician assistant, *ECG* electrocardiogram, FCM flow cytometry, *ICM-9-CM* The International Classification of Diseases, Ninth Revision, Clinical Modification, *LBBB* left bundle branch block, *NSTEMI* non-ST-segment elevation myocardial infarction, *PCI* percutaneous coronary intervention, *STEMI* ST-segment elevation myocardial infarction, *VMS* safety management system.

### Outcome measures

#### Data quality

To investigate data quality (availability, accessibility and completeness), we assessed whether or not particular time points involved in the various definitions were recorded in each of the hospitals. If the data were recorded, the researcher noted how they were accessible. Accessibility was divided into three categories: (1) automatically accessible, (2) partly automatically accessible or (3) manually accessible [3]. Automatically accessible meant that data elements stored within the hospital information system could be easily reviewed (‘only a few mouse clicks away’) and extracted by means of computerised search algorithms. Partly automatically accessible meant that data elements were available in the hospital information system and could be reviewed easily, but could not be extracted by means of a computerised search algorithm and that manual actions were required. Manually accessible meant that data elements were available but only through intense data handling such as paper-based medical record reviews. Additionally, two chart abstractors retrospectively noted per hospital where and in what form data were found, such as in medical records, nurse records, discharge letters, electrocardiograms, procedure letters, correspondence with other health care professionals and in paper form, scanned or in the hospital information system. Finally, we assessed the completeness of the available information at the patient level. We measured the percentage of patients for whom all time points that should be recorded were indeed available.

#### Influence of definitions on indicator scores

To investigate the influence of the performance indicator definition on the scores, we calculated the percentage of patients for whom the treatment delay indicator was below the threshold for each hospital according to the different definitions.

## Results

### Patient data

Secondary data were used from two university hospitals and five tertiary teaching hospitals performing PCI. The bed capacity in these hospitals ranged between 400 to over 1100. Initially, 4471 records were reviewed for inclusion. After excluding records of patients who were not diagnosed with STEMI or excluded based on exclusion criteria (*n* = 3454), 1017 records were available for analyses, ranging between 112 and 236 included records per hospital.

### Outcome measures

#### Data quality

The chart abstractors reported that some hospitals recorded all the required data elements for the calculation of the performance indicator scores. Moreover, automated access to these data was not possible in most cases. The most common ways to access the data were manual or partly automated access (four of the seven hospitals). Fully automated access was not available for any of the data elements, illustrating that data collection was time consuming and costly.

For all available and accessible data, we noted where this information was found (Table 2). For the extraction of data elements with partly automated or manual access, the chart abstractors had to review a combination of medical records, nurse records, discharge letters, electrocardiograms (ECG), procedure letters, correspondence with other health care professionals, and in paper form, scanned or in hospital information system. Table 2 illustrates that the accessibility of data did not only differ per hospital, but also per time point within hospitals.

**Table 2:** Data accessibility per hospital.

	1	2	3	4	5	6	7
First contact	Care pathway registration/patient record (paper)	HIS	HIS	Scanned in HIS/ cardiology department database	Care pathway registration/patient record (paper)/report in HIS	HIS	Patient record (paper)
ECG	Patient record (paper)	Scanned in HIS/cathlab system	Scanned in HIS	Scanned in HIS/cardiology department database	Patient record (paper)/scanned in HIS/care pathway registration	Scanned in separate folder on hard disk	Patient record (paper)/cathlab system
Arrival PCI centre	Patient record (paper)	HIS	HIS	HIS/cardiology department database	Admission system	Separate database	Patient record (paper)/HIS
Sheath insertion	Cathlab report	Separate cathlab system	HIS	Cathlab report in HIS, or cardiology department database	Cathlab report/care pathway registration	Cathlab report in HIS	Cathlab system
First intervention	Cathlab report	Not available	HIS	Cardiology department database	Care pathway registration	Cathlab system	Cathlab system

*HIS* hospital information system.

**Fig. 3:**
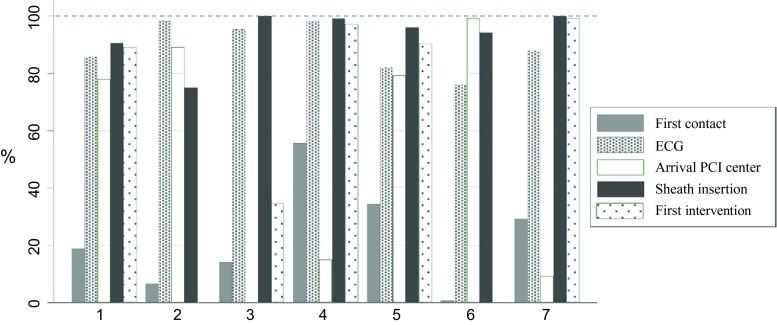
Completeness of time points per hospital.

The completeness of the available information is illustrated in Fig. 3. In 24 % of patients the time of first contact was recorded, in 88 % of the patients the time of ECG, in 51 % of patients the time of arrival at the PCI centre, in 94 % of patients the time of sheath insertion and in 64 % of patients the time of first intervention was recorded. Thus, hospitals vary greatly in completeness of recording, particularly with respect to the time of first contact.

#### Influence of definition on indicator scores

Table 3 shows the percentage of patients satisfying the indicator threshold for each of the definitions and each of the hospitals. Indicator score B was reported best, with 15–50 % missing data across hospitals. Missing data on indicator scores A, C and D were generally over 50 % ranging from 21 to 100 %. When calculable, indicator scores ranged from 57 to 100 % within a given hospital, dependent on the indicator definition.

**Table 3:** Time to PCI indicator: % of patients with missing data and number of times 90 min indicator was reached (*n yes; n total*) per definition per hospital.

Hospital (number)	1	2	3	4	5	6	7	Total (patient with missing data % and indicator reached *n*)
Definition of treatment delay								
A. Dutch ‘VMS safety management program’ guideline	82 % *13; 23*	93 % n/a	86 % 10; 16	44 % 36; 63	69 % 40; 56	99 % n/a	71 % 49; 69	85 % 156; 236
B. Adjusted Dutch ‘VMS safety management program’ guideline	36 % 81; 104	15 % 102; 117	23 % 86; 107	38 % 69; 109	30 % 119; 136	50 % 70; 98	30 % 165; 207	32 % 692; 878
C.^a^ Mean door-to-needle time (IGZ)	*91 %* *11* *(n = 98)*	*85 %* *18* *(n = 106)*	*100 %* *n/a* *(–)*	*87 %* *9* *(n = 15)*	*86 %* *24* *(n = 137)*	*83 %* *23* *(n = 131)*	*93 %* *17* *(n = 22)*	*50 %* *19* *(n = 509)*
D. Door-to-balloon time (ACC/AHA)	24 % 97; 98	100 % n/a	100 % n/a	86 % 16; 16	21 % 135; 136	100 % n/a	91 % 21; 22	74 % 269; 272
**Total (** *n* **of patients)**	127	120	112	112	171	139	236	1017

*IGZ* Dutch Health Care Inspectorate, *n/a* data for indicator not available or fewer than 10 cases

^a^Indicator asks for mean door-to-needle time.

## Discussion

This study illustrates that hospital performance indicator scores for the treatment delay performance indicator are largely incomparable, without laborious manual review.

Three reasons contribute to this incomparability. First, definitions vary for treatment delay performance indicators across the literature, which leads hospitals to vary in the extent to which different time points are recorded and/or used for calculating performance indicators. These differences are also due to the low number of patients and missing data. This is partly due to the choices hospitals make regarding which times to record, but also due to the format in which organisations compel hospitals to report indicators (as percentage or mean). To compare indicator definitions among patients with all data points would be a methodologically sound method. In practice, information is not available for all the data points in any of the patients, as hospitals use different definitions for treatment delay and vary greatly in the extent to which the necessary data are available, accessible and complete. So, this leads to substantially different indicator scores, especially between definitions A and B versus D. Second, the chart abstractors reported that some hospitals had all the required data elements for calculation of the performance indicators and data could not be retrieved easily in any of the hospitals. Moreover, data accessibility not only varied between hospitals, but also between data elements within hospitals. The same hospital could therefore have a relatively low indicator score following one definition and relatively a high score following another definition. Third, we found large variations between hospitals in completeness of time records.

Previous studies on the comparability of medical data in the Netherlands and across Europe similarly showed that required data elements for performance indicators were generally poorly available, accessible and incomplete [3, 16, 17, 18]. This may partly be due to the enormous number of indicators hospitals have to report on for external quality control. In order to compare indicator scores among hospitals it is thus necessary to standardise definitions and record data uniformly, and possibly reduce the number of indicators that hospitals have to accurately measure [19, 20].

To obtain structured data, predefined computer-based forms to record relevant procedures and findings in a structured, standardised format, have been shown to be advantageous [21]. One way to convert the currently used open text into a more structured format is the use of Natural Language Processing tools. However, as most tools are developed in English, further research is required on how to handle the Dutch. Moreover, to enhance the correctness of data items and thus efficiency of secondary use of data, the Netherlands Federation of University Medical Centres is detailing how to best apply the ‘collect once—use many times’ principle [22]. A next step could be to automatically extract data quality items from the hospital information system, checked by a responsible party and submitted to quality registers or other authorised parties [20]. Ideally, data that are in standard codes from comprehensive controlled clinical terminologies such as SNOMED CT can be reused automatically. In the Netherlands, an action plan was recently developed to create a standardised continuity of care record for Dutch hospitals and to create semantically sound subsets of terminologies using SNOMED CT and ICD 10 [20]. Moreover, the USA initiated a nationwide taskforce Meaningful Use of Complex Medical Data in order to overcome problems analysing large amounts of medical data in a timely fashion [23]. Today, hospital performance data can be linked to national mortality databases to provide information on long-term outcomes and survival, provided data can be tracked across providers, which is facilitated by unique person identifiers [24]. Such a national registry is not available for acute coronary syndromes in the Netherlands, whereas this has been possible for many years in other countries, such as Sweden and the UK [25]. Given these advances, performance indicators based on administrative data could be a very useful tool to flag possibilities for quality improvement in hospitals. The extent of these propositions, however, does not provide practitioners with a direct, simple solution. The proposed statements include steps which need to be taken in order to prevent incomparability in the future. Hospital associations in the Netherlands are now working on these steps. Despite the lack of solutions, we feel it is important to inform practice of the critical notion that hospital performance indicator scores for the treatment delay performance indicator are largely incomparable, without laborious manual review.

Our study has several limitations. The time points extracted to calculate indicator scores per hospital may be an overestimation of data completeness compared with indicator scores calculated and supplied by hospitals themselves, because data were extracted by chart abstractors who went to great lengths to obtain data. Moreover, the data obtained by our chart abstractors may deviate from hospital data as the chart abstractors made decisions to clarify which data were necessary to calculate performance indicator scores, such as manually checking all diagnoses in the discharge letter based on the diagnosis and procedure codes. Also, the presence of researchers collecting data on site and the provision of feedback of performance may have influenced documentation of times and performance indicator scores. However, as the patient safety program for which the data were primarily collected was designed to improve guideline adherence and provide hospitals with feedback of their own performances, it would not be appropriate to withhold this information. Consequently, another limitation is the secondary use of data that were obtained for the goal of measuring guideline adherence. For example, the exclusion of uninformative charts means that data were preselected on their quality. In spite of these limitations, our results show that the comparability of indicator scores is influenced by data quality issues.

## Conclusion

In sum, hospitals use different definitions of this one particular quality indicator and vary greatly in the extent to which the necessary data are actually available, accessible and complete, impeding comparability between hospitals. It is important to increase awareness among developers, users and producers of performance indicators regarding the impact of variations in indicator definitions and data quality on indicator scores.

## References

[1] Brook RH, McGlynn EA, Cleary PD (1996). Measuring quality of care. N Engl J Med.

[2] Khan NA, McGilchrist M, Padmanabhan S (2013). Data quality tool. Deliverable of the TRANSFoRm project (Translational Research and Patient Safety in Europe).

[3] Anema HA, Kievit J, Fischer C (2013). Influence of hospital information systems, indicator data collection and computation on reported Dutch hospital performance indicator scores. BMC Health Serv Res.

[4] Kringos DS, Anema HA, ten Asbroek AHA, et al. Beperkt Zicht. Onderzoek naar de betrouwbaarheid, validiteit en bruikbaarheid van prestatie-indicatoren over de kwaliteit van de Nederlandse ziekenhuiszorg. [Research on the reliability, validity and usefullness of performance indicators on the quality of Dutch hospital care]. Amsterdam: AMC; 2012.

[5] Mainz J (2003). Defining and classifying clinical indicators for quality improvement. Int J Qual Health Care.

[6] Williams SC, Watt A, Schmaltz SP (2006). Assessing the reliability of standardized performance indicators. Int J Qual Health Care.

[7] Wollersheim H, Hermens R, Hulscher M (2007). Clinical indicators: development and applications. Neth J Med.

[8] Steg PG, James SK, Atar D, et al. ESC Guidelines for the management of acute myocardial infarction in patients presenting with ST-segment elevation: the Task Force on the management of ST-segment elevation acute myocardial infarction of the European Society of Cardiology (ESC). Eur heart J. 2012;33:2569–619. 10.1093/eurheartj/ehs215 22922416

[9] O’Gara PT, Kushner FG, Ascheim DD, American College of Cardiology Foundation/American Heart Association Task Force on Practice Guidelines (2013). 2013 ACCF/AHA guideline for the management of ST-elevation myocardial infarction: a report of the American College of Cardiology Foundation/American Heart Association Task Force on Practice Guidelines. Circulation.

[10] Boersma E, Primary Coronary Angioplasty vs. Thrombolysis Group (2006). Does time matter? A pooled analysis of randomized clinical trials comparing primary percutaneous coronary intervention and in-hospital fibrinolysis in acute myocardial infarction patients. Eur Heart J.

[11] Postma S, Dambrink JH, de Boer MJ (2014). The influence of residential distance on time to treatment in ST-elevation myocardial infarction patients. Neth Heart J.

[12] Cannon CP, Gibson CM, Lambrew CT (2000). Relationship of symptom-onset-to-balloon time and door-to-balloon time with mortality in patients undergoing angioplasty for acute myocardial infarction. JAMA.

[13] De Luca G, Suryapranata H, Ottervanger JP (2004). Time delay to treatment and mortality in primary angioplasty for acute myocardial infarction: every minute of delay counts. Circulation.

[14] Tra J, Engel J, van der Wulp I (2014). Monitoring guideline adherence in the management of acute coronary syndrome in hospitals: design of a multicentre study. Neth Heart J.

[15] IGZ. Basisset kwaliteitsindicatoren ziekenhuizen [Dutch Health Inspection. Quality indicators for hospitals]. 2012. http://www.igz.nl/Images/Basisset%20kwaliteitsindicatoren%20ziekenhuizen%202012–2_tcm294–305782.pdf. pp. 86–9. Accessed 26 february 2015

[16] Krumholz HM, Anderson JL, Bachelder BL (2008). ACC/AHA. 2008 performance measures for adults with ST-elevation and non–ST-elevation myocardial infarction. Circulation.

[17] Anema HA, van der Veer SN, Kievit J (2014). Influences of definition ambiguity on hospital performance indicator scores: examples from The Netherlands. Eur J Public Health.

[18] Groene O, Kristensen S, Arah OA, DUQuE Project Consortium (2014). Feasibility of using administrative data to compare hospital performance in the EU. Int J Qual Health Care.

[19] Stroetmann VN (Ed.), Kalra D, Lewalle P, et al. Semantic Interoperability for Better Health and Safer Healthcare. Deployment and Research Roadmap for Europe. European Communities, European Commission, Information Society and Media Directorate-General, Belgium 2009.

[20] NFU. Kwaliteitsevaluatie door verbeterde zorg documentatie. [Netherlands Federation of University medical centers. Quality evaluation by improved care registration]. Utrecht: NFU; 2013.

[21] Dentler K, Cornet R, ten Teije A (2014). Influence of data quality on computed Dutch hospital quality indicators: a case study in colorectal cancer surgery. BMC Med Inform DecMak.

[22] Cimino JJ (2007). Collect once, many: enabling the reuse of clinical data through controlled terminologies. J AHIMA.

[23] Blumenthal D, Tavenner M (2010). The ‘meaningful use’ regulation for electronic health records. N Engl J Med.

[24] Herrett E, Shah AD, Boggon R (2013). Completeness and diagnostic validity of recording acute myocardial infarction events in primary care, hospital care, disease registry, and national mortality records: cohort study. BMJ.

[25] Chung S-C, Gedeborg R, Nicholas O (2014). Acute myocardial infarction: a comparison of short-term survival in national outcome registries in Sweden and the UK. Lancet.

